# Identification of trace mineral and nutrient deficiencies as an adjunct to treatment of eating disorders

**DOI:** 10.1186/2050-2974-1-S1-P10

**Published:** 2013-11-14

**Authors:** Jeremy Johnson, Robert Barron, Leanne Barron

**Affiliations:** 1 University of Queensland, Australia; 2 Charles Sturt University, Australia; 3 Brisbane City 6 Day Medical Centre, Brisbane, Australia

## 

Biochemical data from 90 patients presenting to a general practice over the previous five years has been analysed to identify specific nutrient deficiencies.

It is a common complaint amongst families of eating disorder (ED) patients that on first presentation to their GP "the bloods were normal so the GP said that there wasn't anything wrong". This factor may contribute to delayed treatment of the ED, and hence specific nutrient testing at initial presentation may be invaluable.

Routine bloods collected by the GP included FBC, ELFTs, phosphate, iron studies, zinc, magnesium, manganese, B12 , red cell folate, ionised calcium and vitamin D. Whilst many results were noted to be in the low end of the "normal" range, significant deficiencies were most commonly noted in zinc and manganese (95%). In patients with bulimia, of course, hypokalaemia was a significant and often life threatening finding.

Patients were receptive to identification of specific abnormalities, and were then more amenable to being referred on to dietitians, psychologists and psychiatrists to address the eating disorder thus identified.

This data suggests the value of targeted specific tests in ED patients presenting in General Practice, and should encourage patients and doctors to seek more than "basic bloods" at first presentation.

